# A simple approximation algorithm for the diameter of a set of points in an Euclidean plane

**DOI:** 10.1371/journal.pone.0211201

**Published:** 2019-02-08

**Authors:** Jieying Hong, Zhipeng Wang, Wei Niu

**Affiliations:** 1 ESSEC Asia-Pacific, ESSEC Business School, Singapore, Singapore; 2 École Centrale Pékin, Beihang University, Beijing, China; 3 Beijing Advanced Innovation Center for Big Data and Brain Computing, Beihang University, Beijing, China; IUMPA - Universitat Politecnica de Valencia, SPAIN

## Abstract

Approximation algorithms with linear complexities are required in the treatments of big data, however, present algorithms cannot output the diameter of a set of points with arbitrary accuracy and near-linear complexity. By introducing the partition technique, we introduce a very simple approximation algorithm with arbitrary accuracy *ε* and a complexity of *O*(*N* + *ε*^−1^ log *ε*^−1^) for the cases that all points are located in an Euclidean plane. The error bounds are proved strictly, and are verified by numerical tests. This complexity is better than existing algorithms, and the present algorithm is also very simple to be implemented in applications.

## Introduction

Given a finite set of points *T* in a 2D Euclidean plane $R^{2}$, its diameter, denoted by *d*_*T*_, is defined as the maximum distance between two points of *T*. Computing the diameter of a point set is a fundamental problem in computer science. It has been proved that in an Euclidean plane, finding the accurate diameter of a set of *N* points can be reduced to formulating the convex hull of them, with a lower bound of complexity *O*(*N* log *N*) [1–4].

In the science of big data, this classical problem encounters new challenges. For big data, the number of points *N* can be huge, and one usually expects linear or sub-linear algorithms to replace the *O*(*N* log *N*) complexity. Clearly, as *O*(*N* log *N*) is the lower bound, they will certainly be approximate algorithms. In the present paper we only consider the algorithms without pre-processing. For these cases, no sub-linear algorithm can guarantee the accuracy of the approximate diameter, as listing the points will require a minimum *O*(*N*) complexity. Therefore, if we want to obtain an estimable approximate diameter, a linear complexity should be the lower bound.

As an introduction, we here show an easiest approximate algorithm. Given an arbitrary point *p*_*i*_, this algorithm simply reports its maximum distance to other points, *i.e*, $\max_{i,j\in ⟦1,N⟧,j\neq i}\bar{T_{i}T_{j}}$ with $\bar{T_{i}T_{j}}$ the distance between points *T*_*i*_,*T*_*j*_ ∈ *T*, as the approximate diameter *d*_*a*_. It is simply to show that 1 ≤ *d*_*T*_/*d*_*a*_ ≤ 2, implying a very low accuracy of the approximation.

There exist two references for improving this approximation to higher accuracy. Egecioglu and Kalantari designed an algorithm that in *m* iterations the reported approximate diameter *d*_*m*_ satisfies that $1\leq d_{T}/d_{m}\leq \sqrt{5-2\sqrt{3}}\approx 1.24$ [5]. Recently, Alipour *et al*. improved this algorithm to allow fewer iterations, however, the accuracy of the approximation did not change [6].

Another type of approximation problems allows introducing an arbitrary positive number 0 < *ε* < 1 aims at outputting an approximate diameter *d*_*o*_ in linear time, such that

$$\begin{array}{c} 1-\varepsilon \leq \frac{d_{T}}{d_{o}}\leq 1+\varepsilon . \end{array}$$ (1)

Note that this is equivalent to the description that $1\leq \frac{d_{T}}{\left(1-\varepsilon \right)d_{o}}\leq \frac{1+\varepsilon }{1-\varepsilon }$, indicating that if Eq (1) is satisfied, we can output $d_{o}^{*}=\left(1-\varepsilon \right)d_{o}$ instead of $d_{o}^{*}$ to satisfy that

$$\begin{array}{c} 1\leq \frac{d_{T}}{d_{o}^{*}}\leq \frac{1+\varepsilon }{1-\varepsilon }, \end{array}$$ (2)

which is formally consistent to literature. These problems are usually named as (1 + *ε*)-approximations. In two dimensions, lots of approximation algorithms with various near-linear complexities have been developed. Table 1 gives a comparion of different (1 + *ε*)-approximation algorithms to compute the diameter of *T*, in chronological order. However, we remark that most of these algorithms are difficult to be implemented in practices since they require complicated calculations and designs in computational geometry. In this paper, we will introduce a very simple algorithm to approach a near-linear complexity of *O*(*N* + *ε*^−1^ log *ε*^−1^) which is much simpler to be implemented in applications.

**Table 1:** Comparasion of different (1 + *ε*) − *approximation* algorithms to compute the diameter of *T*.

*Author*	*complexity*	*main* *method*
*Agarwal* 1992 [7]	*O*(*ε*^−2^ *N*)	*Random* − *sampling*
*Barequet* 1999 [8]	*O*(*N* + *ε*^−4^)	*Rounding* *to* *Grid*
*Chan* 2000 [9]	*O*(*N* + *ε*^−3^)	*Rounding* *to* *Grid* + *Cones*
*Chan* 2000 [9]	*O*(*N* + *ε*^−2^)	*Grid* + *Cones* + *Dimension* *Reduction*
*Arya* 2014 [10]	*O*(*N* + *ε*^−1^ *N*^1/2^)	*ϵ* − *Dependencies*
*Our* *algorithm*	*O*(*N* + *ε*^−1^ log *ε*^−1^)	*Partition* *technique*

## Approximation algorithm for the diameter in an Euclidean plane

**Fig 1:**
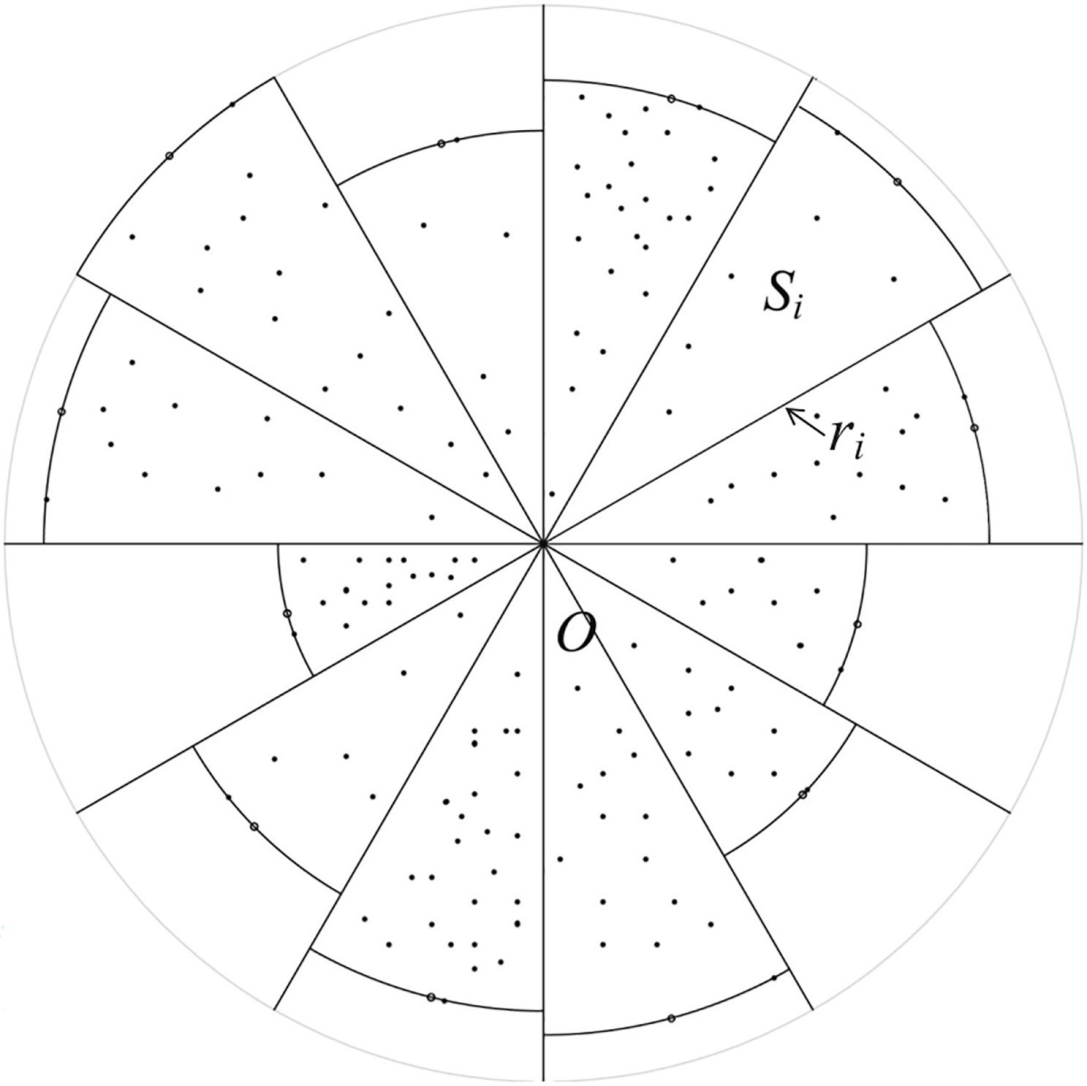
Diagram of the partition for the point set. Solid points are real points in the set *T*, while empty points are virtual points in the set of *p*_*i*_s.

For the finite set of points *T* in $R^{2}$, we choose a point *O* ∈ *T* arbitrarily as the origin, and then divide the plane into 6*n* same regions with $n\in Z_{+}^{*}$. In each region *S*_*i*_ (*i* = 1, …, 6*n*), we can find a farthest point from the origin, and let *r*_*i*_ denote the distance between the farthest point of *S*_*i*_ and the origin. By using the origin *O* as the the center of a circle and *r*_*i*_ as the radius, we can obtain 6*n* sector regions, as Fig 1. We remark that the number of the regions, 6*n*, will allow in the following parts to provide analytical error estimation. Let *p*_*i*_ (*i* = 1, …, 6*n*) be the midpoint of the arc of each sector region, and compute the largest distance *d*_*p*_ of these 6*n* midpoints *p*_*i*_s. Then we propose the following main theorem of this paper which shows the relationship between the diameter *d*_*T*_ of the point set *T* and the largest distance *d*_*p*_. Here we note that the virtual points *p*_*i*_s can be different with the real points in *T*.

**Theorem 1**. *d_T_ is the diameter of a finite set of points T in* $R^{2}$, *and d_p_ is the largest distance of the* 6*n virtual midpoints p_i_s as defined above, then the following statement holds*:

$$\begin{array}{c} \frac{1}{\sqrt{2(1+\cos\frac{(2n-1)\pi }{3n})}}\leq \frac{d_{T}}{d_{p}}\leq \sqrt{2(1+\cos\frac{(2n-1)\pi }{3n})}. \end{array}$$ (3)

In the following we will give the proof of Theorem 1 in two parts: to prove the upper and lower bounds of $\frac{d_{T}}{d_{p}}$ respectively.

### Lower bound of $\frac{d_{T}}{d_{p}}$

**Fig 2:**
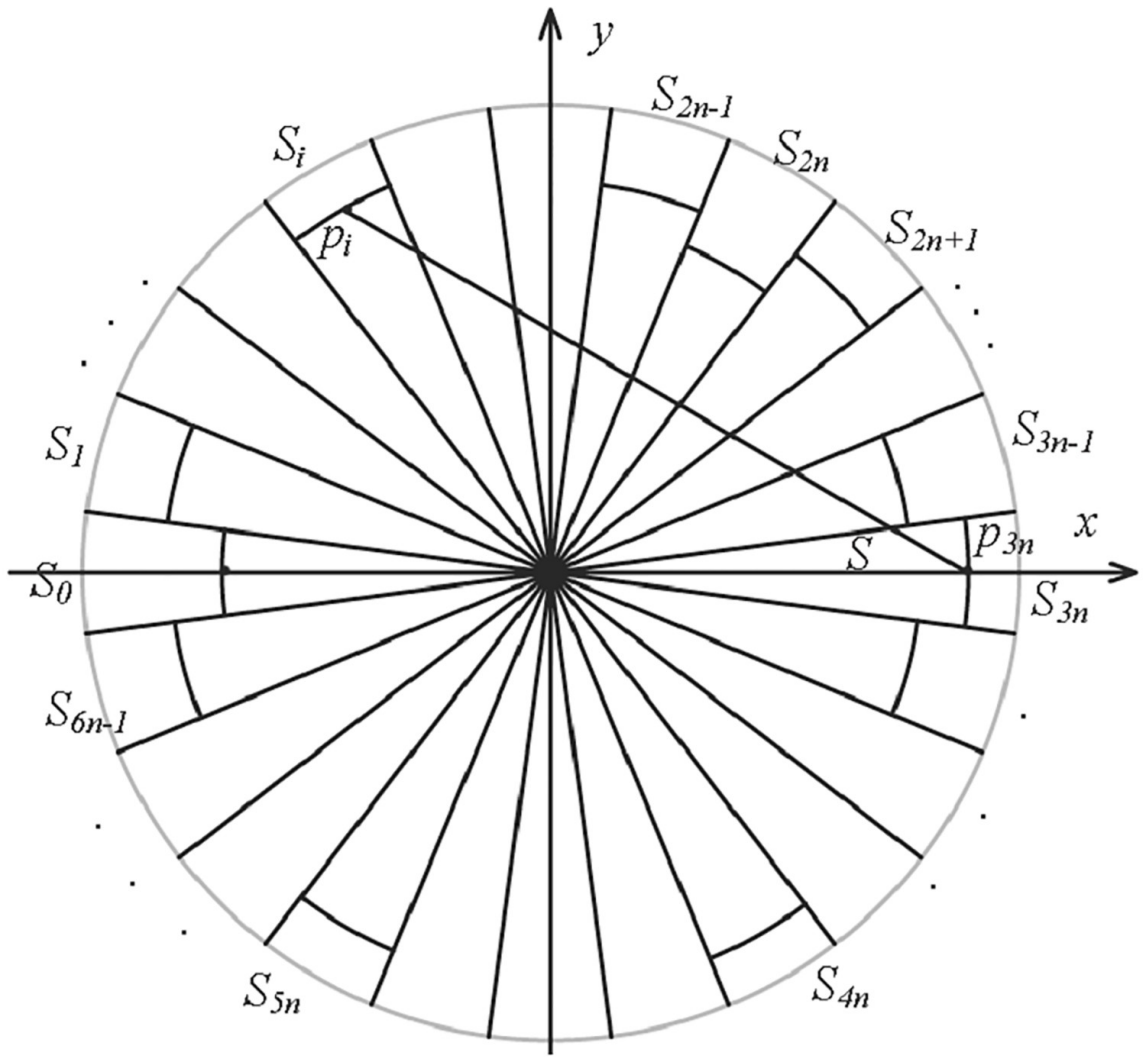
Diagram of the 6*n* regions for the point set and the Cartesian coordinate system.

Without loss of generality, suppose that an endpoint of the line segment *d*_*p*_ is in the region *S*, and then we denote the opposite angle region by *S*_0_ and denote the other regions clockwise by *S*_1_, …, *S*_6*n*−1_. Note that in this way the region *S* is exactly the region *S*_3*n*_. Let the line passing through the origin *O* and the midpoint of the arc of the region *S* be the x-axis, then we can set up the Cartesian coordinate system in the plane, as Fig 2. The coordinate of the midpoint *p*_*i*_ of the arc in each region *S*_*i*_ is $\left(-r_{i}\cos\frac{i\pi }{3n},r_{i}\sin\frac{i\pi }{3n}\right)$, where *i* = 1, …, 6*n* − 1, and *r*_*i*_ is the radius of the sector region *S*_*i*_. Before giving the proof on the lower bound of $\frac{d_{T}}{d_{p}}$, we bring out the following lemmas.

**Lemma 1**. *If an endpoint of the line segment d_p_ is in the region S* (*i.e.*, *S*_3*n*_) *as we supposed above, then the other endpoint of d_p_ cannot be obtained in the regions S*_2*n*+1_, …, *S*_3*n*−1_.

*Proof*. Denote *R* = max(*r*_0_, …, *r*_6*n*−1_), then the relationship *R* ≤ *d*_*p*_ is obvious. For point $p_{i}(-r_{i}\cos\frac{i\pi }{3n},r_{i}\sin\frac{i\pi }{3n})$ in the region *S*_2*n*+1_, …, *S*_3*n*−1_ (i.e., *i* ∈ ⟦2*n* + 1, 3*n* − 1⟧), and the point *p*_3*n*_(−*r*, 0) in the region *S*, we can compute the distance between these two points:

$$\begin{array}{c} {\bar{p_{i}p_{3n}}}^{2}=r^{2}+r_{i}^{2}+2r_{i}r\cos\frac{i\pi }{3n}. \end{array}$$ (4)

Obviously, ${\bar{p_{i}p_{3n}}}^{2}<r^{2}+r_{i}^{2}-rr_{i}$.

Let $f\left(r\right)=r^{2}+r_{i}^{2}-rr_{i}$, then *f*′(*r*) = 2*r* − *r*_*i*_ can be easily obtained. Since *r*, *r*_*i*_ ∈ [0, *R*], then we have that *f*′(*r*) < 0 when *r* ∈ [0, *r*_*i*_/2), and *f*′(*r*) > 0 when *r* ∈ (*r*_*i*_, *R*]. Thus *f*(*r*)_max_ = *f*(0) or *f*(*R*).

When *r* = 0, $f{\left(r\right)}_{\max}=f\left(0\right)=r_{i}^{2}\leq R^{2}$; and when $r=R,f{\left(r\right)}_{\max}=f\left(R\right)=R^{2}+r_{i}^{2}-Rr_{i}$. Let $g\left(r_{i}\right)=f\left(R\right)=R^{2}+r_{i}^{2}-Rr_{i}$, since *r*_*i*_ ∈ [0, *R*], we can obtain the maximum of *g*(*r*_*i*_): *g*(*r*_*i*_)_max_ = *g*(0) or *g*(*R*), and *g*(0) = *g*(*R*) = *R*^2^. Thus *g*(*r*_*i*_) ≤ *R*^2^.

Therefore, ${\bar{p_{i}p_{3n}}}^{2}<r^{2}+r_{i}^{2}-rr_{i}\leq R^{2}$, and the equality arrives when (*r* = *R*, *r*_*i*_ = 0) or (*r* = *R*, *r*_*i*_ = *R*) or (*r*_*i*_ = *R*, *r* = 0). In this way, we can prove that ${\bar{p_{i}p_{3n}}}^{2}<R^{2}\leq d_{p}^{2}$. That means the distance $\bar{p_{i}p_{3n}}\;\left(i=2n+1,\ldots 3n-1\right)$, is always less than *d*_*p*_.

According to the symmetry of the regions, the following lemma can be easily obtained.

**Lemma 2**. *If an endpoint of the line segment d_p_ is in the region S* (*i.e.*, *S*_3*n*_), *then the other endpoint of d_p_ cannot be obtained in the regions S*_3*n*+1_, …, *S*_4*n*−1_.

Moreover, the cases for an endpoint of *d*_*p*_ in the regions *S*_0_, *S*_6*n*−1_, …, *S*_4*n*_ are equivalent to those in the regions *S*_0_, *S*_1_, …, *S*_2*n*_. Therefore, if we suppose that an endpoint of the line segment *d*_*p*_ is in the region *S*, then from Lemma 1 and 2, we only need to consider the 2*n* + 1 cases where the other endpoint of *d*_*p*_ is in the regions *S*_0_, …, *S*_2*n*_. In what follows we will consider two cases in order to compute the lower bound of $\frac{d_{T}}{d_{p}}$.

**Case I**: *i* ∈ ⟦0, 2*n* − 1⟧

As we supposed above, an endpoint of *d*_*p*_ is in the region *S*, then there certainly exists a point *q*_1_ on the arc of the region *S*. The coordinate of *q*_1_ is (*r* cos *θ*, *r* sin *θ*), where $\theta \in [-\frac{\pi }{6n},\frac{\pi }{6n}]$ and *r* is the radius of the sector region *S*. If the other endpoint of *d*_*p*_ is in the region *S*_*i*_ (*i* = 0, 1, …, 2*n* − 1), then there certainly exists a point *q*_2_ on the arc of the region *S*_*i*_ and the coordinate of *q*_2_ is (−*r*_*i*_ cos *θ*_*i*_, *r*_*i*_ sin *θ*_*i*_), where $\theta_{i}\in \left[\frac{i\pi }{3n}-\frac{\pi }{6n},\frac{i\pi }{3n}+\frac{\pi }{6n}\right]$ and *r*_*i*_ is the radius of the sector region *S*_*i*_.

The distance between the two points *q*_1_ and *q*_2_ can be computed as

$$\begin{array}{c} \begin{array}{ccc} {\bar{q_{1}q_{2}}}^{2} & = & {\left(r_{i}\cos\theta_{i}+r\cos\theta \right)}^{2}+{\left(r_{i}\sin\theta_{i}-r\sin\theta \right)}^{2}\\ & = & r_{i}^{2}+r^{2}+2r_{i}r\cos\left(\theta_{i}+\theta \right), \end{array} \end{array}$$ (5)

where $\theta_{i}+\theta \in \left[\frac{(i-1)\pi }{3n},\frac{(i+1)\pi }{3n}\right]$, for *i* = 0, 1, …, 2*n* − 1. Since cos *x* is monotone decreasing in $\left[0,\frac{2\pi }{3}\right]$, we have $\cos\left(\theta_{i}+\theta \right)\geq \cos\frac{(i+1)\pi }{3n}$. Thus

$$\begin{array}{c} {\bar{q_{1}q_{2}}}^{2}\geq r_{i}^{2}+r^{2}+2r_{i}r\cos\frac{(i+1)\pi }{3n}. \end{array}$$ (6)

From the definition of *d*_*T*_, we know that

$$\begin{array}{c} d_{T}\geq \bar{q_{1}q_{2}}\geq \sqrt{r_{i}^{2}+r^{2}+2r_{i}r\cos\frac{(i+1)\pi }{3n}}. \end{array}$$ (7)

Since $d_{p}=\bar{p_{i}p_{3n}}=\sqrt{r^{2}+r_{i}^{2}+2r_{i}r\cos\frac{i\pi }{3n}}$, the following relationship can be obtained.

$$\begin{array}{c} \frac{d_{T}}{d_{p}}\geq \sqrt{\frac{r^{2}+r_{i}^{2}+2rr_{i}\cos\frac{(i+1)\pi }{3n}}{r^{2}+r_{i}^{2}+2rr_{i}\cos\frac{i\pi }{3n}}}. \end{array}$$ (8)

From (8), we have

$$\begin{array}{c} \frac{d_{T}}{d_{p}}\geq \sqrt{1+\frac{2rr_{i}\left(\cos\frac{(i+1)\pi }{3n}-\cos\frac{i\pi }{3n}\right)}{r^{2}+r_{i}^{2}+2rr_{i}\cos\frac{i\pi }{3n}}}=\sqrt{1+\frac{2(\cos\frac{(i+1)\pi }{3n}-\cos\frac{i\pi }{3n})}{\frac{r}{r_{i}}+\frac{r_{i}}{r}+2\cos\frac{i\pi }{3n}}}. \end{array}$$ (9)

When *x* ∈ [0, 2*n* − 1], the function $\cos\frac{\pi x}{3n}$ is monotone decreasing, thus $\cos\frac{(i+1)\pi }{3n}-\cos\frac{i\pi }{3n}<0$. In addition, $\frac{r}{r_{i}}+\frac{r_{i}}{r}\geq 2$, Then we can obtain that

$$\begin{array}{c} \frac{d_{T}}{d_{p}}\geq \sqrt{1+\frac{2(\cos\frac{(i+1)\pi }{3n}-\cos\frac{i\pi }{3n})}{2+2\cos\frac{i\pi }{3n}}}=\sqrt{\frac{1+\cos\frac{(i+1)\pi }{3n}}{1+\cos\frac{i\pi }{3n}}}. \end{array}$$ (10)

Let $f\left(x\right)=\frac{1+\cos\frac{(x+1)\pi }{3n}}{1+\cos\frac{x\pi }{3n}}$ and *x* ∈ [0, 2*n* − 1]. We consider the monotonicity of *f*(*x*) and compute its derivative for this purpose.

$$\begin{array}{c} f^{\prime }\left(x\right)=\frac{\frac{\pi }{3n}\left[-\sin\frac{(x+1)\pi }{3n}\left(1+\cos\frac{x\pi }{3n}\right)+\sin\frac{x\pi }{3n}\left(1+\cos\frac{(x+1)\pi }{3n}\right)\right]}{{\left(1+\cos\frac{x\pi }{3n}\right)}^{2}}. \end{array}$$ (11)

Since the denominator of *f*′(*x*) is always greater than 0, we only consider the sign of its numerator. Let *g*(*x*) be the numerator of *f*′(*x*) divided by *π*/(3*n*).

$$\begin{array}{c} \begin{array}{ccc} g(x) & = & -\sin\frac{(x+1)\pi }{3n}\left(1+\cos\frac{x\pi }{3n}\right)+\sin\frac{x\pi }{3n}\left(1+\cos\frac{(x+1)\pi }{3n}\right)\\ & = & \sin\frac{x\pi }{3n}-\sin\frac{(x+1)\pi }{3n}+\sin\left(\frac{x\pi }{3n}-\frac{(x+1)\pi }{3n}\right)\\ & = & \sin\frac{x\pi }{3n}-\sin\frac{(x+1)\pi }{3n}-\sin\frac{\pi }{3n}. \end{array} \end{array}$$ (12)

The derivative of *g*(*x*) is $\frac{\pi }{3n}\left(\cos\frac{x\pi }{3n}-\cos\frac{(x+1)\pi }{3n}\right)$. In the case *x* ∈ [0, 2*n* − 1], it is obviously that *g*′(*x*) > 0, and we can get that *g*(0) < 0 and *g*(2*n* − 1) < 0 by computation. Thus for any *x* ∈ [0, 2*n* − 1], *g*(*x*) < 0 and also *f*′(*x*) < 0. Therefore we know that *f*(*x*) is monotone decreasing in the interval [0, 2*n* − 1].

All this leads up to the following inequality:

$$\begin{array}{c} \frac{d_{T}}{d_{p}}\geq \sqrt{\frac{1+\cos\frac{(i+1)\pi }{3n}}{1+\cos\frac{i\pi }{3n}}}\geq \sqrt{f(2n-1)}=\frac{1}{\sqrt{2(1+\cos\frac{(2n-1)\pi }{3n})}}. \end{array}$$ (13)

**Case II**: *i* = 2*n*

In this case $d_{p}=\bar{p_{2n}p_{3n}}=\sqrt{r^{2}+r_{i}^{2}-r_{i}r}$, and from the proof of Lemma 1 we know that $\sqrt{r^{2}+r_{i}^{2}-r_{i}r}\leq R$. Moreover, since *d*_*T*_ ≥ *R*, we have

$$\begin{array}{c} \frac{d_{T}}{d_{p}}\geq \frac{R}{\sqrt{r_{i}^{2}+r^{2}-rr_{i}}}\geq \frac{R}{R}=1>\frac{1}{\sqrt{2(1+\cos\frac{(2n-1)\pi }{3n})}}. \end{array}$$ (14)

Concluding the two cases, we can obtain the lower bound of $\frac{d_{T}}{d_{p}}$:

$$\begin{array}{c} \frac{d_{T}}{d_{p}}\geq \frac{1}{\sqrt{2(1+\cos\frac{(2n-1)\pi }{3n})}}. \end{array}$$ (15)

### Upper bound of $\frac{d_{T}}{d_{p}}$

In this subsection, we will prove the upper bound of $\frac{d_{T}}{d_{p}}$. Similar to the approach for the proof of the lower bound, supposing that an endpoint of the line segment *d*_*T*_ is in the region *S*, and then we only need to consider the cases that the other endpoint of *d*_*T*_ is in the region *S*_*i*_ for *i* ∈ ⟦0, 2*n*⟧.

**Case I**: *i* ∈ ⟦1, 2*n*⟧

As we supposed above, an endpoint of *d*_*T*_ is in the region *S* and the other endpoint is in the region *S*_*i*_, which denoted by *m*_1_ and *m*_2_ respectively. The coordinates of *m*_1_ and *m*_2_ are ($-\hat{r}\cos\theta ,\hat{r}\sin\theta$) and ($-{\hat{r}}_{i}\cos\theta_{i},{\hat{r}}_{i}\sin\theta_{i}$) respectively, where $\hat{r}\in \left[0,r\right]$, ${\hat{r}}_{i}\in \left[0,r_{i}\right]$, $\theta \in [-\frac{\pi }{6n},\frac{\pi }{6n}]$ and $\theta_{i}\in \left[\frac{i\pi }{3n}-\frac{\pi }{6n},\frac{i\pi }{3n}+\frac{\pi }{6n}\right]$. The distance between the two points $\bar{m_{1}m_{2}}$, is exactly *d*_*T*_ and thus $\bar{m_{1}m_{2}}\geq R$.

Furthermore,

$$\begin{array}{c} \begin{array}{ccc} {\bar{m_{1}m_{2}}}^{2} & = & {\left({\hat{r}}_{i}\cos\theta_{i}+\hat{r}\cos\theta \right)}^{2}+{\left({\hat{r}}_{i}\sin\theta_{i}-\hat{r}\sin\theta \right)}^{2}\\ & = & {\hat{r}}_{i}^{2}+{\hat{r}}^{2}+2{\hat{r}}_{i}\hat{r}\cos\left(\theta_{i}+\theta \right), \end{array} \end{array}$$ (16)

where $\theta_{i}+\theta \in \left[\frac{(i-1)\pi }{3n},\frac{(i+1)\pi }{3n}\right]$, for *i* = 1, …, 2*n*. Since cos *x* is monotone decreasing in [0, *π*], we have $\cos\left(\theta_{i}+\theta \right)\leq \cos\frac{(i-1)\pi }{3n}$. Thus

$$\begin{array}{c} {\bar{m_{1}m_{2}}}^{2}\leq {\hat{r}}_{i}^{2}+{\hat{r}}^{2}+2{\hat{r}}_{i}\hat{r}\cos\frac{(i-1)\pi }{3n}. \end{array}$$ (17)

Let $h\left(\hat{r}\right)={\hat{r}}^{2}+{\hat{r}}_{i}^{2}+2a\hat{r}{\hat{r}}_{i}$, where $a=\cos\frac{(i-1)\pi }{3n}\in \left(-\frac{1}{2},1\right]$.

1. In the case $a=\cos\frac{(i-1)\pi }{3n}\in \left[0,1\right]$, $h\left(\hat{r}\right)={\hat{r}}^{2}+{\hat{r}}_{i}^{2}+2a\hat{r}{\hat{r}}_{i}\leq r^{2}+r_{i}^{2}+2arr_{i}$. And since $d_{p}\geq \bar{p_{i}p_{3n}}=\sqrt{r^{2}+r_{i}^{2}+2rr_{i}\cos\frac{i\pi }{3n}}$, we know that
  $$\begin{array}{c} \frac{d_{T}}{d_{p}}\leq \frac{\sqrt{r^{2}+r_{i}^{2}+2rr_{i}\cos\frac{(i-1)\pi }{3n}}}{\sqrt{r^{2}+r_{i}^{2}+2rr_{i}\cos\frac{i\pi }{3n}}}. \end{array}$$ (18)
2. In the case $a=\cos\frac{(i-1)\pi }{3n}\in \left(-\frac{1}{2},0\right)$, we can compute that $h^{\prime }\left(\hat{r}\right)=2\left(\hat{r}+a{\hat{r}}_{i}\right)$.
  - If ${\hat{r}}_{i}\geq 2r$, then $h^{\prime }\left(\hat{r}\right)\leq 0$ when $\hat{r}\in \left[0,r\right]$. And we can get the maximum of $h(\hat{r})$: $h{\left(\hat{r}\right)}_{\max}=h\left(0\right)={\hat{r}}_{i}^{2}\leq r_{i}^{2}\leq R^{2}$. Thus $d_{T}=\bar{m_{1}m_{2}}\leq R$, and as we mentioned above, $\bar{m_{1}m_{2}}\geq R$, those lead that
    $$\begin{array}{c} d_{T}=\bar{m_{1}m_{2}}=R\;\text{and}\;r_{i}=R,r=0. \end{array}$$ (19)
    In this moment,
    $$\begin{array}{c} d_{p}\geq \bar{p_{i}p_{3n}}=\sqrt{r^{2}+r_{i}^{2}+2rr_{i}\cos\frac{i\pi }{3n}}=R. \end{array}$$ (20)
    Therefore we have
    $$\begin{array}{c} \frac{d_{T}}{d_{p}}\leq \frac{R}{R}=1<\frac{\sqrt{r^{2}+r_{i}^{2}+2rr_{i}\cos\frac{(i-1)\pi }{3n}}}{\sqrt{r^{2}+r_{i}^{2}+2rr_{i}\cos\frac{i\pi }{3n}}}. \end{array}$$ (21)
  - If $0\leq {\hat{r}}_{i}<2r$, then $h^{\prime }\left(\hat{r}\right)\leq 0$ when $\hat{r}\in \left[0,-a{\hat{r}}_{i}\right]$ and $h^{\prime }\left(\hat{r}\right)\geq 0$ when $\hat{r}\in \left[-a{\hat{r}}_{i},r\right]$. In this case, $h{\left(\hat{r}\right)}_{\max}=h\left(0\right)$ or *h*(*r*).
    If $h{\left(\hat{r}\right)}_{\max}=h\left(0\right)$, same as the case (a),
    $$\begin{array}{c} \frac{d_{T}}{d_{p}}\leq 1. \end{array}$$ (22)
    If $h{\left(\hat{r}\right)}_{\max}=h\left(r\right)={\hat{r}}_{i}^{2}+r^{2}+2ar{\hat{r}}_{i}$, then let $t\left({\hat{r}}_{i}\right)={\hat{r}}_{i}^{2}+r^{2}+2ar{\hat{r}}_{i}$, where ${\hat{r}}_{i}\in \left[0,r_{i}\right]$. The derivative of $t({\hat{r}}_{i})$ is $2({\hat{r}}_{i}+ar)$. If ${\hat{r}}_{i}<-ar$, then $t^{\prime }\left({\hat{r}}_{i}\right)<0$, and $t{\left({\hat{r}}_{i}\right)}_{\max}=t\left(0\right)=r^{2}$. In this case, $d_{T}=\bar{m_{1}m_{2}}\leq \sqrt{h(r)}\leq \sqrt{t(0)}=r\leq R$, and then similar to the case (a),
    $$\begin{array}{c} \frac{d_{T}}{d_{p}}\leq 1. \end{array}$$ (23)
    If ${\hat{r}}_{i}\geq -ar$, then $t^{\prime }\left({\hat{r}}_{i}\right)\geq 0$, and $t{\left({\hat{r}}_{i}\right)}_{\max}=t\left(r_{i}\right)=r^{2}+r_{i}^{2}+2arr_{i}$. Here we have $d_{T}\leq \sqrt{h(r)}\leq \sqrt{t(r_{i})}=r^{2}+r_{i}^{2}+2arr_{i}$, and thus
    $$\begin{array}{c} \frac{d_{T}}{d_{p}}\leq \frac{\sqrt{r^{2}+r_{i}^{2}+2rr_{i}\cos\frac{(i-1)\pi }{3n}}}{\sqrt{r^{2}+r_{i}^{2}+2rr_{i}\cos\frac{i\pi }{3n}}}. \end{array}$$ (24)

Since $\frac{\sqrt{r^{2}+r_{i}^{2}+2rr_{i}\cos\frac{(i-1)\pi }{3n}}}{\sqrt{r^{2}+r_{i}^{2}+2rr_{i}\cos\frac{i\pi }{3n}}}>1$, we can summarize all the cases in Case I and get

$$\begin{array}{c} \frac{d_{T}}{d_{p}}\leq \frac{\sqrt{r^{2}+r_{i}^{2}+2rr_{i}\cos\frac{(i-1)\pi }{3n}}}{\sqrt{r^{2}+r_{i}^{2}+2rr_{i}\cos\frac{i\pi }{3n}}}=\sqrt{1+\frac{2(\cos\frac{(i-1)\pi }{3n}-\cos\frac{i\pi }{3n})}{\frac{r}{r_{i}}+\frac{r_{i}}{r}+2\cos\frac{i\pi }{3n}}}. \end{array}$$ (25)

Then by using the similar approach for computing the lower bound of $\frac{d_{T}}{d_{p}}$, where *i* ∈ ⟦1, 2*n*⟧, we can deduce the following inequality

$$\begin{array}{c} \frac{d_{T}}{d_{p}}\leq \sqrt{\frac{1+\cos\frac{(2n-1)\pi }{3n}}{1+\cos\frac{2n\pi }{3n}}}=\sqrt{2(1+\cos\frac{(2n-1)\pi }{3n})}. \end{array}$$ (26)

**Case II**: *i* = 0

In this case, $d_{p}\geq \bar{p_{i}p_{3n}}=r_{i}+r$. Moreover,

$$\begin{array}{c} \begin{array}{ccc} d_{T} & = & \bar{m_{1}m_{2}}=\sqrt{{\hat{r}}_{i}^{2}+{\hat{r}}^{2}+2{\hat{r}}_{i}\hat{r}\cos\left(\theta_{i}+\theta \right)}\;\left(\text{where}\;\theta_{i}+\theta \in \left[-\frac{\pi }{3n},\frac{\pi }{3n}\right]\right)\\ & \leq & \sqrt{{\hat{r}}_{i}^{2}+{\hat{r}}^{2}+2{\hat{r}}_{i}\hat{r}}={\hat{r}}_{i}+\hat{r}\leq r_{i}+r. \end{array} \end{array}$$ (27)

Therefore,

$$\begin{array}{c} \frac{d_{T}}{d_{p}}\leq \frac{r_{i}+r}{r_{i}+r}=1. \end{array}$$ (28)

From Case I and II, the supper bound of $\frac{d_{T}}{d_{p}}$ can be concluded:

$$\begin{array}{c} \frac{d_{T}}{d_{p}}\leq \sqrt{2(1+\cos\frac{(2n-1)\pi }{3n})}. \end{array}$$ (29)

In this way, we have proved the Theorem 1, and the relationship in this theorem can be also written as

$$\begin{array}{c} \frac{1}{\sqrt{2(1+\cos\frac{(2n-1)\pi }{3n})}}\leq \frac{d_{T}}{d_{p}}\leq \sqrt{2(1+\cos\frac{(2n-1)\pi }{3n})}. \end{array}$$ (30)

This theorem therefore provides a fast approximation for the diameter of the point set *T*.

### Remarks

We remark that the complexities of visiting all *N* points in set *T*, calculating their polar coordinates and renewing the values of *r*_*i*_ are all linearly *O*(*N*). The complexity of calculating the diameter of 6*n* virtual points is *O*(*n* log *n*) as introduced in section 1. Indeed, even if we compare all pairs among these points via brute force, the complexity will be at most *O*(*n*^2^), which will be negligible by comparing to *O*(*N*) if *N* is huge. Therefore, we conclude that our approximation algorithm has a complexity of *O*(*N* + *n* log *n*), which is deterministically linear complexity when *N* ≫ *n*.

In addition, recalling the problem descriptions (1) and (2), we can also formulate the outputted diameter and calculate the necessary number of regions *n* for an arbitrary accuracy 0 < *ε* < 1. It is easy to verify that when

$$\begin{array}{c} \begin{array}{cc} n> & \frac{2\pi }{2\pi -3\cos^{-1}\left(\frac{-1+4\varepsilon +2\varepsilon^{2}}{2{\left(\varepsilon -1\right)}^{2}}\right))},\\ d_{o}= & \frac{d_{p}}{\left(1-\varepsilon \right)\sqrt{2(1+\cos\frac{(2n-1)\pi }{3n})}},\\ d_{o}^{*}= & \frac{d_{p}}{\sqrt{2(1+\cos\frac{(2n-1)\pi }{3n})}}, \end{array} \end{array}$$ (31)

Eqs (1) and (2) are both satisfied. For small *ε* Taylor expansion shows that

$$\begin{array}{c} \frac{2\pi }{2\pi -3\cos^{-1}\left(\frac{-1+4\varepsilon +2\varepsilon^{2}}{2{\left(\varepsilon -1\right)}^{2}}\right)}\approx \frac{\pi }{\sqrt{3}}\varepsilon^{-1}, \end{array}$$ (32)

which indicates that

$$\begin{array}{c} n∼\varepsilon^{-1}, \end{array}$$ (33)

and the complexity writes

$$\begin{array}{c} O\left(N+n\log n\right)∼O\left(N+\varepsilon^{-1}\log\varepsilon^{-1}\right). \end{array}$$ (34)

We therefore remark that all values of *d*_*p*_, *d*_*o*_ and $d_{o}^{*}$ can be used as approximate diameters, depending on the accuracy interval one requires.

## Numerical tests

As illustrated in the previous section, expressions (3), (1) and (2) describe the error bounds of *d*_*p*_, *d*_*o*_ and $d_{o}^{*}$ respectively. However, in practice these upper and lower bounds correspond to the worst cases, while for most situations the error will be even smaller. In this section we show by three different point sets this error distribution, respectively. In the first point set *T*_(1)_, the Cartesian coordinate of each point is (*x*, *y*), where *x* and *y* are independent random variables uniformly distributed in [0, 1), leading to a diameter close to the diagonal; in the second point set *T*_(2)_, the polar coordinate of each point is (*r*, *θ*), where *θ* is a random variable homogeneously distributed in [0, *π*), and *r* is a random variable with Gaussian distribution *N*(0, 1) [11]; the third point set *T*_(3)_ is chosen from a real database on the positions of fluid particles [12]. Both *T*_(1)_ and *T*_(2)_ have 1 × 10^6^ discrete points, while *T*_(3)_ have 4 × 10^5^ discrete points. We simply use *O*(*n*^2^) brute force method to calculate the diameter of the 6*n* virtual points. Indeed, this does not yield any inconvenience in the calculation, while the computational time of the case *n* = 100 is only 1.01 times of that of the case *n* = 1. Therefore we can conclude that the calculations are of near-linear complexity.

**Fig 3:**
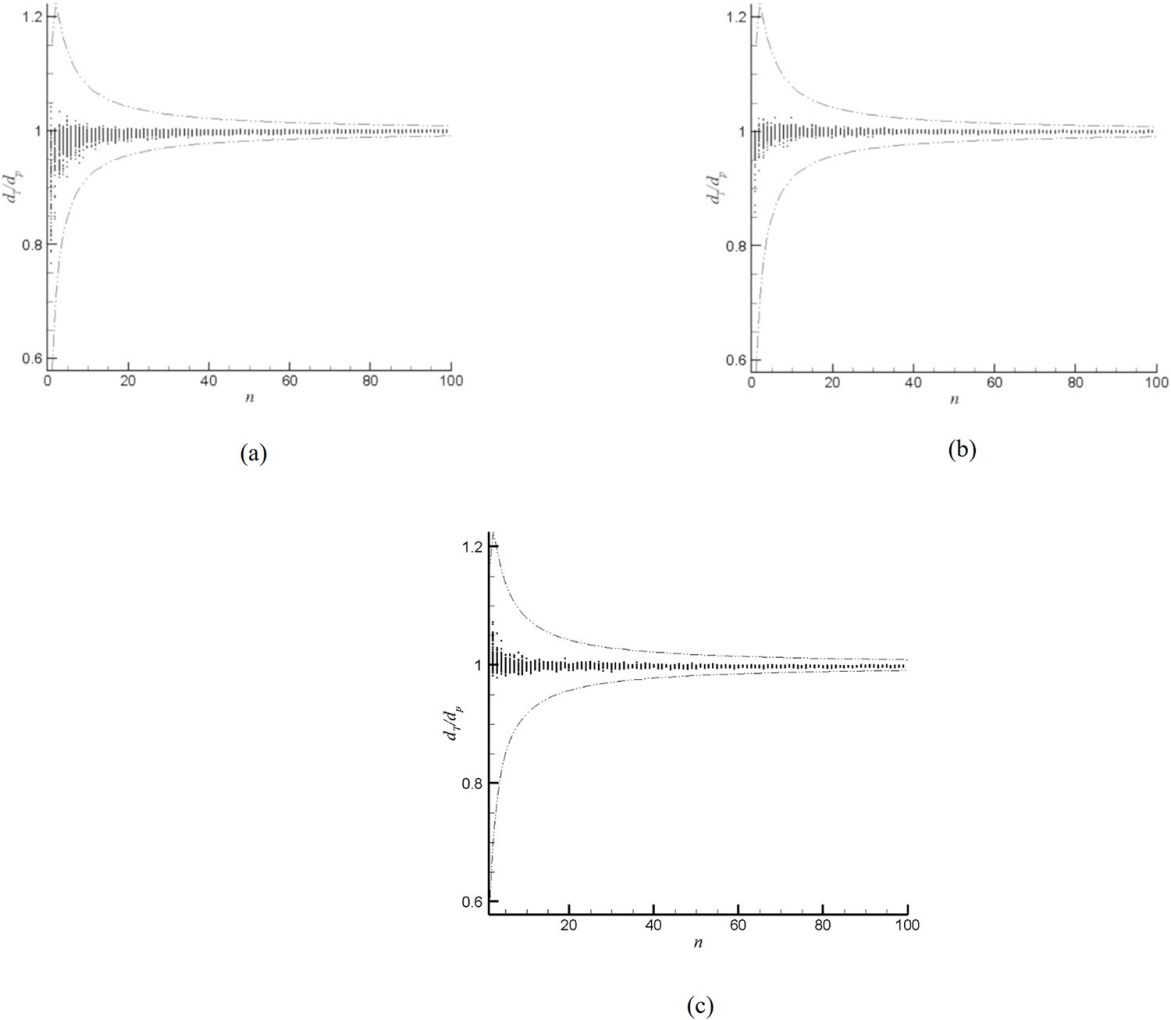
Numerical results of *d*_*T*_/*d*_*p*_. (a) *T*_(1)_ case; (b) *T*_(2)_ case; (c) *T*_(3)_ case. The theoretical error bounds are shown as dash-dotted lines.

Without loss of generality, here we use our algorithm to output the values of *d*_*p*_, and compare them with the theoretical error ranges (3), as shown in Fig 3. We randomly select 100 different origin points for each value of *n* respectively. Clearly, for all cases, most calculated diameters *d*_*p*_ are quite close to the real value *d*_*T*_, which are even quite better than the theoretically worst bounds (shown as dash-dotted lines in Fig 3). These results then show the effectiveness of the present algorithm.

**Fig 4:**
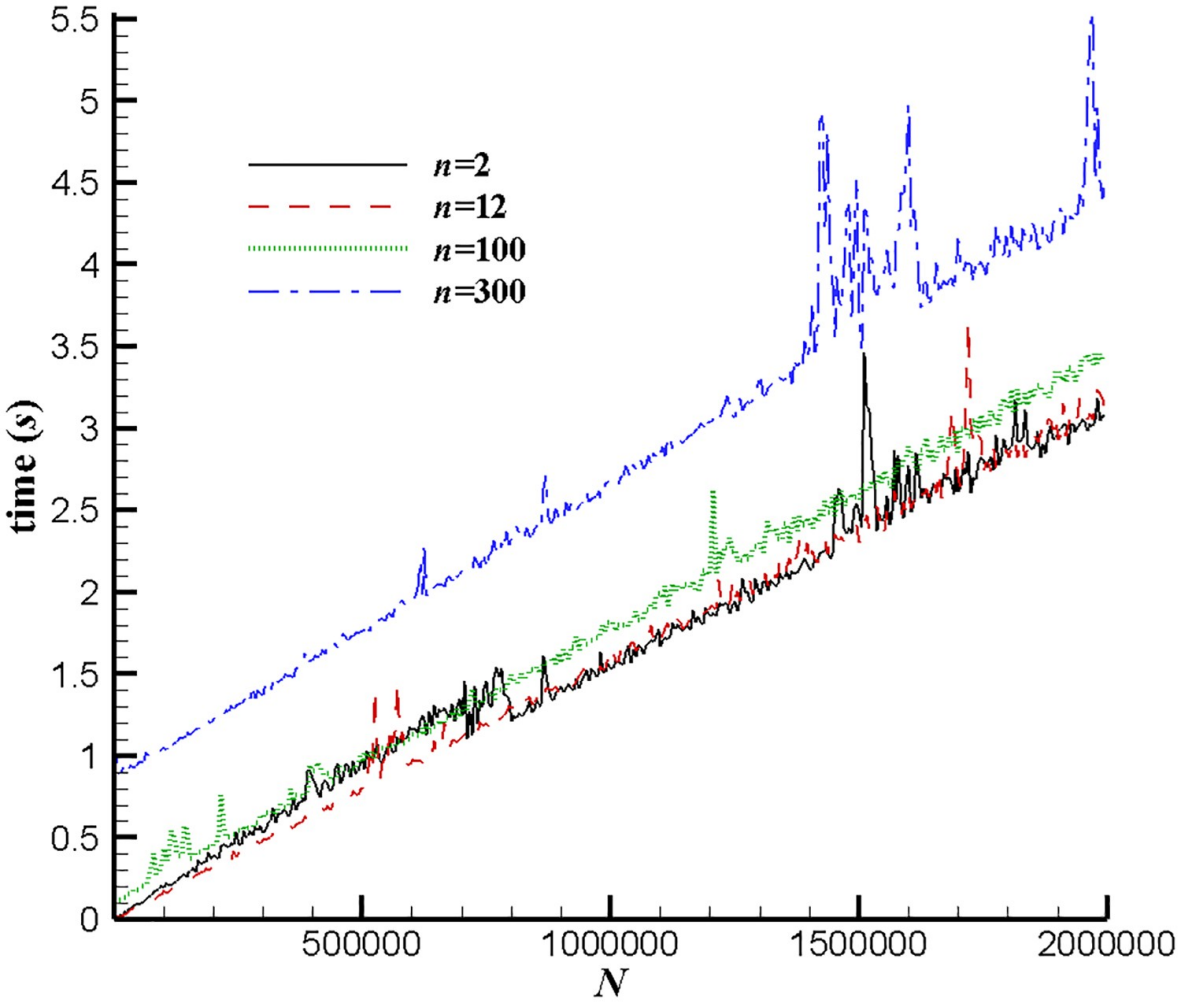
CPU time with different *N* values. Points are generated similarly to the *T*_(1)_ case. *n* = 2, 12, 100 and 300 respectively.

We also present the CPU time in Fig 4. Points are generated similarly to the *T*_(1)_ case, *i.e.*, point coordinates are independent random variables uniformly distributed in [0, 1). The partition parameter *n* is fixed as 2, 12, 100 and 300 respectively. Calculations are performed via single thread at Intel Core i5-6200U CPU 2.30GHz, interpreted by Python 2.5.1 in the IDLE software. Fig 4 shows that the CPU time is linear to the value of *N*, illustrating that the present algorithm is of nearly linear complexity. In addition, although no optimization is implemented to accelerate the calculations, the real performance is acceptable since calculating the approximate diameter of 2 × 10^6^ points with *n* = 100 (corresponding to relative error *ε* = 9 × 10^−7^) only costs about 3 seconds. These evidences suggest the implementation of the present algorithm in real applications.

## Conclusion

As a fundamental problem of big data, linear approximation algorithms for the diameter of a set of points will be potentially useful. By introducing the partition technique, we introduce an approximation algorithm with arbitrary accuracy and deterministically linear complexity. The implementation of this algorithm is very simple and does not require any complicated data structure. Note that the lower bound of the proposed algorithm is *O*(*N* + *n* log *n*) with *n* of the order of *ε*^−1^, while a brute force visiting algorithm for virtual points will increase this to *O*(*N* + *n*^2^). In practice *n* will be much smaller than *N*, therefore *O*(*n*^2^) will be negligible by comparing to *O*(*N*). In addition, increasing the number of partition *n* does not increase any multiple coefficient to *O*(*N*), which indicates the robustness of the near-linear complexity of our algorithm. Comparing to existing approximation algorithms, the present algorithm shows a lowest complexity *O*(*N* + *ε*^−1^ log *ε*^−1^). Also, another advantage of the present algorithm is that it is very simple to be implemented, which does not require any complicated data structure or geometry calculation.

The present contribution is a preliminary attempt in 2D plane. For higher dimensional cases, this method might also be extended, but a division of hyper-sphere [13–15] will be required. In those situations, other partition schemes will be more efficient. For example, one may use high-dimensional Cartesian coordinates instead of the division of hyper-sphere. The related accuracy will also be more complicated, and is expected to be investigated in our future work.
